# Co‐expression of glycosylated aquaporin‐1 and transcription factor NFAT5 contributes to aortic stiffness in diabetic and atherosclerosis‐prone mice

**DOI:** 10.1111/jcmm.14843

**Published:** 2020-01-22

**Authors:** Rosalinda Madonna, Vanessa Doria, Anikó Görbe, Nino Cocco, Péter Ferdinandy, Yong‐Jian Geng, Sante Donato Pierdomenico, Raffaele De Caterina

**Affiliations:** ^1^ Institute of Cardiology University of Pisa Pisa Italy; ^2^ Center of Excellence on Aging and Regenerative Medicine (CeSI‐Met) “G. d'Annunzio” University Chieti Chieti Italy; ^3^ Center for Cardiovascular Biology and Atherosclerosis Research McGovern School of Medicine University of Texas Health Science Center at Houston Houston TX USA; ^4^ Department of Pharmacology and Pharmacotherapy Semmelweis University Budapest Hungary; ^5^ Pharmahungary Group Szeged Hungary; ^6^ Tor Vergata University Hospital Rome Italy; ^7^ Institute of Cardiology “G. d'Annunzio” University Chieti Italy

**Keywords:** arterial stiffening, cytoskeletal remodelling, diabetes, hypercholesterolaemia, hyperosmolarity, subclinical atherosclerosis

## Abstract

Increased stiffness characterizes the early change in the arterial wall with subclinical atherosclerosis. Proteins inducing arterial stiffness in diabetes and hypercholesterolaemia are largely unknown. This study aimed at determining the pattern of protein expression in stiffening aorta of diabetic and hypercholesterolaemic mice. Male Ins^2+/Akita^ mice were crossbred with ApoE^−/−^ (Ins^2+/Akita^: ApoE^−/−^) mice. Relative aortic distension (relD) values were determined by ultrasound analysis and arterial stiffness modulators by immunoblotting. Compared with age‐ and sex‐matched C57/BL6 control mice, the aortas of Ins^2+/Akita^, ApoE^−/−^ and Ins^2+/Akita^:ApoE^−/−^ mice showed increased aortic stiffness. The aortas of Ins^2+/Akita^, ApoE^−/−^ and Ins^2+/Akita^:ApoE^−/−^ mice showed greater expression of VCAM‐1, collagen type III, NADPH oxidase and iNOS, as well as reduced elastin, with increased collagen type III‐to‐elastin ratio**.** The aorta of Ins^2+/Akita^ and Ins^2+/Akita^:ApoE^−/−^ mice showed higher expression of eNOS and cytoskeletal remodelling proteins, such as F‐actin and α‐smooth muscle actin, in addition to increased glycosylated aquaporin (AQP)‐1 and transcription factor NFAT5, which control the expression of genes activated by high glucose‐induced hyperosmotic stress. Diabetic and hypercholesterolaemic mice have increased aortic stiffness. The association of AQP1 and NFAT5 co‐expression with aortic stiffness in diabetes and hypercholesterolaemia may represent a novel molecular pathway or therapeutic target.

## 1 BACKGROUND

Compared with non‐diabetic individuals, diabetic patients suffer more aggressive, rapidly progressive atherosclerosis often with earlier complications, such as arterial stiffness.^1^, ^2^ This is because altered glucose metabolism in diabetes can modify and increase the impact of comorbidities, such as hypercholesterolaemia.^3^ In these patients at high risk of cardiovascular events, the identification of clinical and molecular markers that allow an early diagnosis of atherosclerosis is essential. Arterial stiffening is also an established clinical marker of early subclinical atherosclerosis.^2^, ^4^, ^5^

The arterial stiffness has been shown to be associated with hypertension^6^ and kidney disease.^7^ The underlying mechanisms may be attributed to (a) endothelial dysfunction leading to vasoconstriction; (b) oxidative stress and inflammatory factors;^8^ and (c) vascular calcifications.^9^ However, proteins that promote the arterial stiffening in diabetes and hypercholesterolaemia are less known. Pathogenetic mechanisms and biomarkers of early‐stage vascular abnormalities, such as arterial stiffening, can be identified through animal models of diabetes and hypercholesterolaemia.^10^ Cytoskeletal reorganization and arterial remodelling are adaptive responses to altered biomechanical stress. Reorganization of the vascular cytoskeleton is causally connected with arterial stiffening and contractile dysfunction.^11^ It can be favoured by an increase in wall stress or biomechanical stretching caused by high blood pressure.^11^ In diabetes, arterial remodelling can occur as the result of a chronic increase in wall stress or biomechanical stretching due to high glucose‐induced hyperosmolar stress, often fluctuating in uncontrolled diabetes. We have previously shown that concentrations of glucose up to 30.5 mmol/L, attainable under hyperglycaemic conditions, induce an up‐regulation of F‐actin, α‐smooth muscle actin (ASMA) and cytoskeletal remodelling in human‐induced pluripotent stem (iPS) cells.^12^ These effects appeared to be mediated through an aquaporin isoform 1 (AQP1)‐ and transcription factor nuclear factor of activated T cells 5 (NFAT5)‐dependent hyperosmolar stress which, through this mechanism, promotes cell migration.^12^ Thus, hyperosmolar stress can act as a most important biophysical factor that promotes cytoskeletal remodelling and the migration of vascular cells, such as vascular smooth muscle cells (VSMCs), within the arterial media. Directed migration of VSMCs requires a polarized reorganization of the cytoskeleton actin. From the six mammalian actin genes, the expression of VSMC‐specific cytoskeleton actin ASMA appears to be regulated by both CArG promoter elements, such as the transcriptional coactivator myocardin,^13^ and NFAT5.^14^ NFAT5, a rel/NF‐κB family member, is a transcription factor activated by hyperosmolar stress, which regulates the expression of osmosensing genes, including those involved in cell migration and cytoskeletal remodelling.^12^, ^15^, ^16^, ^17^, ^18^

In the present research, we sought to determine the impact of hypercholesterolaemia and diabetes on arterial wall stiffness in the aortas of genetic diabetes and hypercholesterolaemia mice models. We also analysed gene expression underlying arterial stiffening at the protein level, including the product of genes responsive to biomechanical stretch, such as high glucose‐induced hyperosmolar stress. Understanding changes in protein expression and molecular circuits activated by concomitantly present risk factors, such as hypercholesterolaemia and diabetes, may indicate new targets for diagnostic and therapeutic strategies aimed at early atherosclerosis in high‐risk patients.

## 2 METHODS

### 2.1 Materials

All chemicals were purchased from Sigma St Louis MO, unless otherwise specified.

### 2.2 Experimental animals

Ins2^+/Akita^ heterozygous, C57BL/6J and apoE^−/−^ homozygous mutation (apoE‐knockout) mice were originally obtained from The Jackson Laboratory (Bar Harbor, ME) ref 28, 29. All mice were on a C57BL/6J background. All procedures were approved by the Institutional Ethics Committee for Animal Research (Protocol number 11/2012/CEISA/COM). All animal experiments complied with the ARRIVE guidelines and were carried out in accordance with the United Kingdom Animals (Scientific Procedures) Act, 1986 and associated guidelines, the European Union (EU) Directive 2010/63/EU for animal experiments and the United States of America (USA) National Institutes of Health guide for the care and use of Laboratory animals (NIH Publications No. 8023, revised 1978).

### 2.3 Generation of Ins2^+/Akita^: ApoE^−/−^ mice and study population

Diabetic male *Ins2^+/Akita^: ApoE^+/+^* (Ins^2+/Akita^ heterozygous mice) were crossed with non‐diabetic female *Ins2^+/+Akita^: ApoE* ^−/−^ mice (F0). The resulting F1 generation consisted of heterozygous apoE^±^ (Ins^2+/Akita^:apoE^±^ and Ins^2+/+^:apoE^±^) mice. From this F1 generation, diabetic male Ins^2+/Akita^:apo^E±^ mice were crossed with non‐diabetic female Ins^2+/+^:apoE^−/−^ mice. The resulting F2 generation consisted of homozygous apoE^−/−^ (Ins^2+/Akita^:apoE^−/−^ and Ins^2+/+^:apoE^−/−^) and heterozygous apoE^±^ (Ins^2+/Akita^:apoE^±^ and Ins^2+/+^: apo^E±^) mice. Subsequently, diabetic male Ins2^+/Akita^:apoE^−/−^ mice (from F2 generation) and non‐diabetic female Ins^2+/+^:apoE^−/−^ were set up as breeding pairs to produce an F3 generation of diabetic Ins^2+/Akita^:apoE^−/−^ mice and non‐diabetic control Ins^2+/+^:apoE^−/−^ mice. For the present study, we used male mice from the F3 generation (diabetic and non‐diabetic control) because male Ins2^+/Akita^ mice exhibit a more severe and homogeneous diabetic phenotypes compared with female mice.^19^ The study population comprised male wild‐type C57BL/6 mice (bodyweight: 15 ± 4 g, age: 3 months; n = 3), male Ins2^+/Akita^ diabetic mice (bodyweight: 11 ± 2 g, n = 3), male apoE^−/−^ mice (bodyweight: 21 ± 5 g, n = 3) and male Ins2^+/Akita^:apoE^−/−^ mice (bodyweight: 19 ± 2 g, n = 3). All animals were specific pathogen‐free and kept in a temperature‐controlled environment in a ventilated rack with a 12‐h:12‐h light:dark cycle. Mice had free access to water and standard rodent chow diet (Teklad 2018; Harlan Laboratories), which contains <0.1% cholesterol and fat as 18% of total calories. Genotypes were determined by polymerase chain reaction (PCR) amplification of tail DNA using protocols provided by The Jackson Laboratory. The diabetic phenotype was confirmed in mice at 4‐5 weeks after birth by blood glucose values >250 mg/dL with a hand‐held glucometer (Contour; Bayer Health Care) measured with a drop of blood from tail puncture. The hypercholesterolaemic phenotype was confirmed by total cholesterol values >150 mg/dL. The disease penetrance was 100% in mice with the Ins^2Akita^ mutation.^20^

### 2.4 Biochemical assays

Plasma glucose, total cholesterol and triglyceride concentrations were measured using an enzymatic colorimetric method by Vitros DT60 II Chemistry System (Ortho‐Clinical Diagnostics) according to the manufacturer's instructions (Table 1).

**Table 1:** Phenotypic and biochemical characteristics of Ins^2+^/Akita, ApoE^−/−^ and Ins^2+^/Akita:ApoE^−/−^ mice

	C57/BL6 control	Ins^2+^/Akita	ApoE^−/−^	Ins^2+^/Akita:ApoE^−/−^
Bodyweight, g	30 ± 0.5	22 ± 0.3	35 ± 0.7	26 ± 0.5
Fasted plasma glucose, mg/dL	110 ± 10	350 ± 20*	150 ± 15	390 ± 25*
Fasted plasma total cholesterol, mg/dL	105 ± 5	125 ± 8	425 ± 25*	625 ± 15*, §
Fasted plasma triglyceride, mg/dL	89 ± 7	105 ± 5	95 ± 6	103 ± 10

Note

Data shown are mean ± SD (n = 7 mice/group). To determine plasma glucose and lipid levels, tail blood samples were collected from each group of mice under fasting conditions.

Abbreviation: ApoE, apolipoprotein E.

* *P* < .05 vs C57/BL6 control mice.

^§^ *P* < .05 vs ApoE^−/−^ mice.

### 2.5 Mouse ultrasound imaging

Transthoracic ultrasound imaging on wild‐type C57BL/6 male mice and sex/age‐matched Ins2^+/Akita^, apoE^−/−^ and Ins2^+/Akita^:apoE^−/−^ mice was performed to determine systolic and diastolic aortic diameters. Two‐dimensional and M‐mode echocardiographic images were recorded and analysed using a portable ultrasound apparatus (Esaote) equipped with a 21‐MHz linear probe. Images were obtained in the parasternal long‐axis view. Aortic diameter instantaneous values were derived from B‐mode images and were recorded in late systole and late diastole using edge detection.^21^ Mean diameter (Dm) and relative distension (relD) values (this latter considered as a surrogate marker for arterial stiffness) were evaluated from the obtained diameter waveforms; relD was calculated as (D_s_ − D_d_)/D_d_ and expressed as a percentage (where D_s_ is the diameter in systole and D_d_ the diameter in diastole). After measurements, mice were killed, and their aorta excised for protein extraction.

### 2.6 Immunoblotting

Total proteins from aortas, harvested from wild‐type C57BL/6 male mice and sex/age‐matched Ins2^+/Akita^, apoE^−/−^ and Ins2^+/Akita^:apoE^−/−^ mice, were isolated, electroblotted and incubated with the following primary and secondary antibodies: (a) mouse monoclonal anti‐collagen type III (dilution 1:2000, Sigma Aldrich); (b) mouse monoclonal anti‐elastin (dilution 1:2000, Sigma Aldrich); (c) mouse monoclonal anti‐AQP1 (dilution 1:600, Santa Cruz Biotechnologies); (d) rabbit polyclonal anti‐F‐Actin (dilution 1:500, Santa Cruz); (e) mouse policlonal anti‐eNOS (dilution 1:2500, BD Transduction Laboratories, San Jose, CA); (f) rabbit polyclonal anti‐VCAM‐1 (dilution 1:600, Santa Cruz); (g) rabbit polyclonal anti‐ICAM‐1 (dilution 1:600, Santa Cruz); (h) mouse monoclonal anti‐IL‐1β (dilution 1:200, R&D Systems); (i) mouse monoclonal anti‐TNF‐α (dilution 1:200, R&D Systems); (j) rabbit monoclonal anti‐NADPH oxidase (dilution 1:1000, Abcam); (k) rabbit polyclonal anti‐NFAT5 (dilution 1:600, Santa Cruz); (l) mouse policlonal iNOS (dilution 1:2500, BD Transduction Laboratories); (m) rabbit monoclonal anti‐NFAT5 (dilution 1:600, Santa Cruz); (n) mouse monoclonal anti‐ASMA (dilution 1:2000, Sigma Aldrich); (o) mouse monoclonal anti‐β‐actin (Sigma); and (p) mouse anti‐GAPDH (dilution 1:5000, Ambion), as previously described.^22^

### 2.7 Statistical analysis

Groups were compared by one‐way analysis of variance followed by Scheffé's test for multiple comparisons. Statistical significance was defined as *P* < .05.

## 3 RESULTS

### 3.1 Aortic stiffness

**Figure 1:**
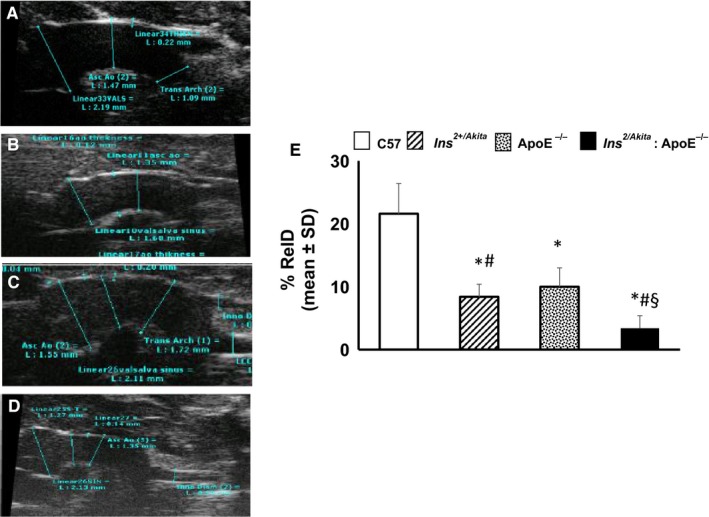
Aortic stiffness. B‐mode image sequence of the aortas was used to measure diameters and distension in Ins^2Akita^, ApoE^−/−^ and Ins^2Akita^: ApoE^−/−^ mice and from sex‐ and age‐matched (3 months old) C57BL/6 non‐diabetic non‐hypercholesterolaemic control mice. A‐D shows representative aorta images from sex‐ and age‐matched (3 months old) C57BL/6 non‐diabetic control mice (A), Ins2^Akita^ mice (B), ApoE^−/−^ mice (C) and Ins2^Akita^: ApoE^−/−^ mice (D). E, The bar graph represents the mean ± SD of each relative distension (relD) values from 3 separate experiments. *P < .05 vs C57BL/6 control mice; #*P* < .05 vs ApoE^−/−^ mice; §*P* < .05 vs Ins^2Akita^ mice and ApoE^−/−^ mice; N = 8 mice/group

To develop a murine model that can be used to study the role of diabetes and hypercholesterolaemia in aortic stiffness and in its protein expression, diabetic hypercholesterolaemic male Ins2^+/Akita^:apoE^−/−^ mice and non‐diabetic hypercholesterolaemic female Ins^2+/+^:apoE^−/−^ were crossed to produce diabetic hypercholesterolaemic Ins^2+/Akita^:apoE^−/−^ mice (Table 1). Three‐month‐old apoE^−/−^ and Ins2^+/Akita^ mice had significantly lower relative distension (relD) values than non‐diabetic non‐hypercholesterolaemic control mice (Figure 1). The combination of the two comorbidities further reduced aortic distension: compared with Ins^2+/Akita^ mice and apoE^−/−^ mice, relD values were more than halved in Ins2^+/Akita^:apoE^−/−^ mice (Figure 1).

### 3.2 Structural proteins

**Figure 2:**
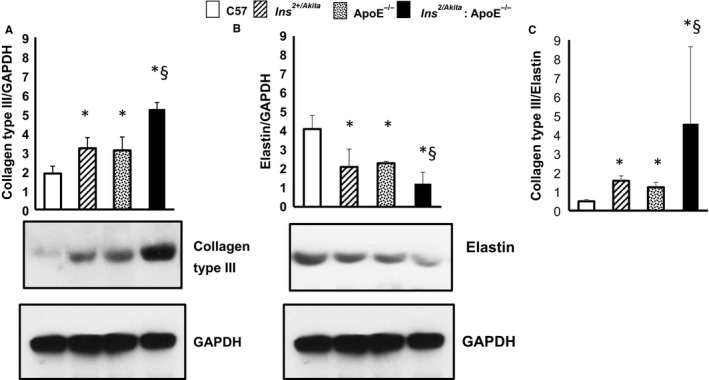
Expression of collagen type III and elastin. Western analysis of (A) collagen type III, (B) elastin expression and (C) collagen type III/elastin ratio in aortas from Ins2^Akita^, ApoE^−/−^ and Ins2^Akita^: ApoE^−/−^ mice and from sex‐ and age‐matched (3 months old) C57BL/6 non‐diabetic control mice, with GAPDH serving as a loading control. The bar graph represents for each value the mean ± SD from 3 separate experiments. *P < .05 vs C57BL/6 control mice; #*P* < .05 vs ApoE^−/−^ mice; §*P* < .05 vs Ins2^Akita^ mice and ApoE^−/−^ mice; N = 8 mice/group

Collagen and elastin are the main structural proteins related to aortic stiffness. Abnormalities in the quantity and quality of collagen and elastin contribute to aortic stiffening.^23^ The aortas of 3‐month‐old apoE^−/−^ and Ins2^+/Akita^ mice had significantly higher levels of collagen type III (Figure 2A) and lower levels of elastin (Figure 2B), with increased collagen type III‐to‐elastin ratio (Figure 2C) than non‐diabetic, non‐hypercholesterolaemic control mice. The combination of the two comorbidities further modified the expression of these proteins and their ratio, as levels of collagen type III and elastin were higher and lower in Ins2^+/Akita^:apoE^−/−^ mice compared with Ins^2+/Akita^ mice and apoE^−/−^ mice, respectively (Figure 2).

### 3.3 VCAM‐1

**Figure 3:**
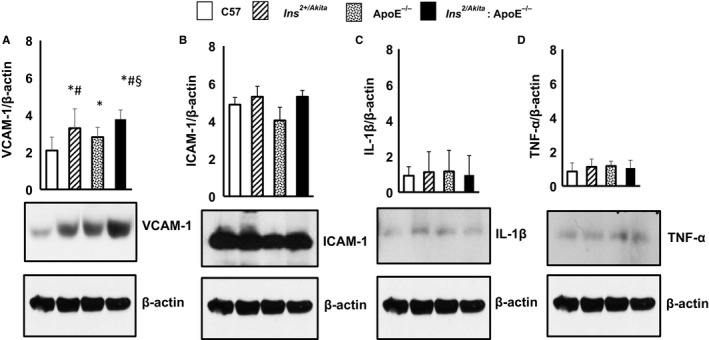
Expression of VCAM‐1, ICAM‐1, IL‐1β and TNF‐α. Western analysis of (A) VCAM‐1 (B) ICAM‐1, (C) IL‐1β and (D) TNF‐α expression in aortas from Ins2^Akita^, ApoE^−/−^ and Ins2^Akita^: ApoE^−/−^ mice and from sex‐ and age‐matched (3 months old) C57BL/6 non‐diabetic control mice, with β‐actin serving as a loading control. The bar graph represents for each value the mean ± SD from 3 separate experiments. *P < .05 vs C57BL/6 control mice; #*P* < .05 vs ApoE^−/−^ mice; §*P* < .05 vs Ins2^Akita^ mice and ApoE^−/−^ mice; N = 8 mice/group

An increased expression of pro‐inflammatory cytokines and adhesion molecules has been suggested to contribute to collagen quality abnormalities, which can destabilize collagen fibres in the vascular wall.^24^, ^25^ While there were no apparent changes in the expression of monocyte chemotactic protein‐1, intercellular adhesion molecule (ICAM‐1), tumour necrosis factor α and interleukin‐1 in the aortas of apoE^−/−^ and Ins2^+/Akita^ mice (Figure 3B‐D), the expression of vascular cell adhesion molecule (VCAM)‐1 was significantly higher in Ins2^+/Akita^ mice and apoE^−/−^ mice than in non‐diabetic non‐hypercholesterolaemic control mice (Figure 3A). The combination of the two comorbidities further increased the expression of VCAM‐1, as the levels of this early pro‐inflammatory adhesion protein were higher in Ins2^+/Akita^:apoE^−/−^ mice compared with Ins^2+/Akita^ mice and apoE^−/−^ mice (Figure 3A).

### 3.4 Nitric oxide synthase and NADPH oxidase

**Figure 4:**
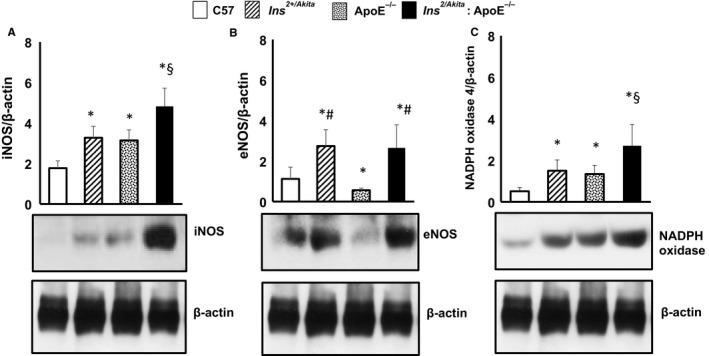
Expression of iNOS, eNOS and NADPH oxidase. Western analysis of (A) iNOS, (B) eNOS and (C) NADPH oxidase expression in aortas from Ins2^Akita^, ApoE^−/−^ and Ins2^Akita^: ApoE^−/−^ mice and from sex‐ and age‐matched (3 months old) C57BL/6 non‐diabetic control mice, with β‐actin serving as a loading control. The bar graph represents for each value the mean ± SD from three separate experiments. *P < .05 vs C57BL/6 control mice; #*P* < .05 vs ApoE^−/−^ mice; §*P* < .05 vs Ins2^Akita^ mice and ApoE^−/−^ mice; N = 8 mice/group

Increased formation of reactive oxygen species (ROS), including peroxynitrite and nitrotyrosine, may contribute to abnormalities in the quality and destabilization of collagen fibres in the vascular wall.^24^, ^25^ NAD(P)H oxidases produce O_2_^−^ and play a major role in ROS generation and oxidative stress. Increased nitric oxide (NO) synthesis and reduced NO bioavailability, which leads to an increase in the NO pool, have been demonstrated in diabetes.^26^, ^27^ Such excess of NO can cause the formation of peroxynitrite and nitrotyrosine. The aortas of 3‐month‐old apoE^−/−^ and Ins2^+/Akita^ mice had significantly higher levels of iNOS and NADPH oxidase than non‐diabetic non‐hypercholesterolaemic control mice (Figure 4A). The combination of diabetes and hypercholesterolaemia further increased the expression of iNOS and NADPH oxidase, as the levels of these proteins were higher in Ins2^+/Akita^:apoE^−/−^ mice compared with Ins^2+/Akita^ mice and apoE^−/−^ mice (Figure 4A). Interestingly, only the aorta of Ins^2+/Akita^ mice and Ins^2+/Akita^:ApoE^−/−^ mice showed greater expression of eNOS (Figure 4B), suggesting that the up‐regulation of both iNOS and eNOS may contribute to the increased NO pool in diabetic animal models with chronic hyperglycaemia.

### 3.5 Cytoskeletal remodelling and expression of AQ1 and NFAT5

**Figure 5:**
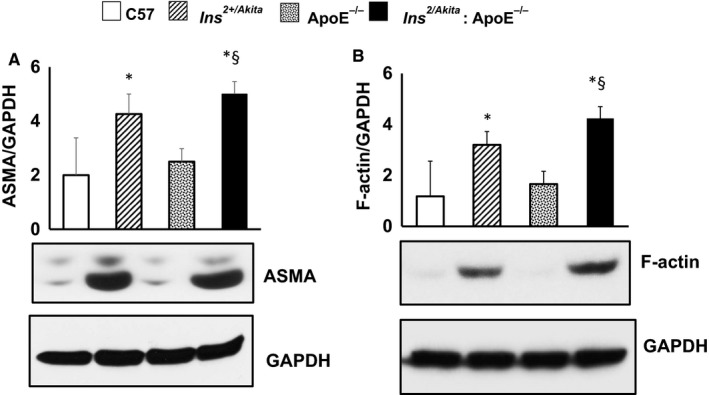
Expression of smooth muscle α‐actin and F‐actin. Western analysis of (A) smooth muscle α‐actin (ASMA) and (B) F‐actin expression in aortas from Ins2^Akita^, ApoE^−/−^ and Ins2^Akita^: ApoE^−/−^ mice and from sex‐ and age‐matched (3 months old) C57BL/6 non‐diabetic control mice, with GAPDH serving as a loading control. The bar graph represents for each value the mean ± SD from three separate experiments. *P < .05 vs C57BL/6 control mice; §*P* < .05 vs Ins2^Akita^ mice and ApoE^−/−^ mice; N = 8 mice/group

**Figure 6:**
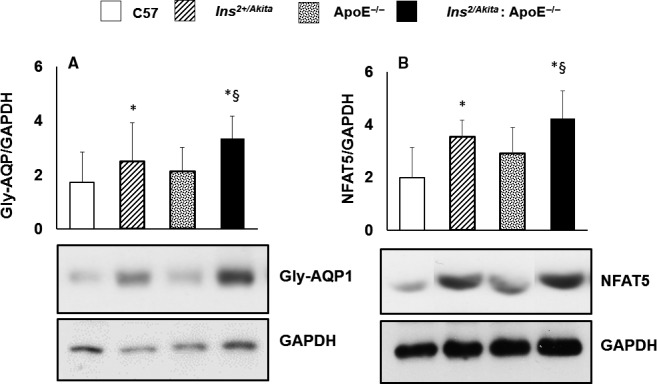
Expression of glycosylated AQP1 isoform and NFAT5. Western analysis of glycosylated AQP1 isoform and NFAT5 expression in aortas Ins^2Akita^, ApoE^−/−^ and Ins^2Akita^: ApoE^−/−^ mice and from sex‐ and age‐matched (3 months old) C57BL/6 non‐diabetic control mice, with GAPDH serving as a loading control. The bar graph represents for each value the mean ± SD from three separate experiments. *P < .05 vs C57BL/6 control mice and ApoE^−/−^ mice; §*P* < .05 vs Ins^2Akita^ mice and ApoE^−/−^ mice; N = 8 mice/group

Arterial stiffening depends on cytoskeletal remodelling besides the accumulation of collagen. Cytoskeletal remodelling requires a polarized reorganization of cytoskeletal actin, which involves the switch from globular actin (G‐actin) to filamentous actin (F‐actin) and the increased synthesis of VSMC‐specific cytoskeletal ASMA. AQP1 and NFAT5 are key proteins that regulate cellular homeostasis and the expression of hypertonicity‐responsive genes, including the cytoskeleton‐remodelling proteins F‐actin and ASMA. In our murine model of diabetes and hypercholesterolaemia, only the aorta of Ins^2+/Akita^ mice and Ins^2+/Akita^: ApoE^−/−^ mice featured greater expressions of ASMA (Figure 5A) and F‐actin (Figure 5B). In parallel, the aorta of Ins^2+/Akita^ mice and Ins^2+/Akita^: ApoE^−/−^ mice showed a higher expression of glycosylated AQP1 (Figure 6A) and NFAT5 (Figure 6B).

## 4 DISCUSSION

The current study demonstrates, for the first time in literature, that diabetic hypercholesterolaemic Ins^2+/Akita^: ApoE^−/−^ mice are characterized by increased arterial stiffness, in a manner associated with up‐regulation of hypertonicity‐responsive factors, such as AQ1 and NFAT5, along with genes implicated in early inflammation and atherosclerosis, such as VCAM‐1; cytoskeletal remodelling, such as F‐actin and ASMA; endothelial dysfunction, such as iNOS and eNOS; and ROS generation, such as NADPH oxidase. These findings suggest that (a) diabetes and hypercholesterolaemia add on and perhaps synergize in inducing aortic stiffening; (b) the biomechanical stretch due to hyperglycaemia‐induced hyperosmolar stress is specifically associated with aortic stiffening in diabetes; and (c) the hypertonicity‐responsive transcription factor NFAT5 may play a key role in cytoskeletal remodelling and the subsequent arterial stiffening in diabetes.

Several factors can determine the increase in arterial stiffness, including decreased synthesis of elastin, increased collagen content and altered organization of elastic and collagen fibres.^30^, ^31^ In the present study, we analysed the expression of collagen type III and elastin, which are the main structural proteins in the aortic wall, using Western analysis. In the aortas of diabetic and hypercholesterolaemic mice, we observed quantitative changes in the collagen and elastin contents, associated with the up‐regulation of early pro‐inflammatory and pro‐atherogenic molecules, such as VCAM‐1, and increased expression of nitric oxide synthases iNOS and eNOS, and NADPH oxidase. VCAM‐1 is an early pro‐inflammatory molecule that plays a key pro‐atherogenic role in macrophage infiltration in the aortic wall.^32^ The up‐regulation of iNOS and eNOS leads to increased NO pool, with consequent formation of peroxynitrite and nitrotyrosine and increased oxidative stress in the aortic wall.^26^, ^27^ The up‐regulation of NADPH oxidases contributes to increased oxidative stress. Inflammation and oxidative stress have been shown to cause abnormalities in the quality of collagen fibres and a disarray of elastic fibres, due to elastolysis, which in turn impairs the elastic properties and the structural integrity of the aortic wall, leading to arterial stiffening.^33^ Our results imply that diabetes and hypercholesterolaemia can both contribute to arterial stiffening at least in part through inflammation and increased oxidative stress in the aortic wall, leading to abnormalities in the quality of elastic and collagen fibres. These effects might be aggravated in ageing, although in our study we did not analyse the influence of ageing as a comorbidity.

Here, we provide the first evidence that NFAT5 and AQP1 are involved in the hypertonicity‐related induction of arterial stiffening, since NFAT5 and AQP1 were increased in the aortas of diabetic Ins^2+/Akita^ mice exposed to high glucose‐induced hyperosmolar stress, independent of the presence of hypercholesterolaemia. AQP1 is a water channel protein expressed widely in vascular endothelia and smooth muscle cells, where it increases cell membrane water permeability, as well as cell motility and migration, and regulates cellular homeostasis during osmolarity changes.^12^, ^34^ NFAT5 is a transcription factor that regulates cellular homeostasis in response to hyperosmolar stress.^15^, ^35^, ^36^, ^37^, ^38^ Furthermore, NFAT5 regulates cell migration and the expression of cytoskeletal proteins, including actins, in several cell types, including VSMCs 14 and iPS cells under high glucose‐induced hyperosmolar conditions.^12^ Thus, in conditions of increased wall stress, such as arterial hypertension 11, 39 or high glucose‐induced hyperosmolar stress (as shown in the present study), NFAT5 contributes to the transition of VSMCs in the arterial media from quiescence to a motility state. These changes, together with wall thickening, characterize vascular remodelling and arterial stiffening,^11^, ^40^ and are controlled by a wide range of transcription factors, such as AP‐1, serum response factor and myocardin.^41^, ^42^ In this context, we revealed that the hyperosmolar stretch‐stimulated aortic wall responds by enhancing protein abundance of NFAT5, in line with observations made by other research groups.^43^ Our results suggest that cytoskeletal remodelling is a specific mechanism of aortic stiffening in diabetes, with AQP1 and NFAT5 as main candidates involved in the specific cell signalling leading to an up‐regulation of cytoskeleton‐remodelling proteins.

We acknowledge some limitations of our study. First, ours is a cross‐sectional study; thus, proteins that have been shown to be modulated by diabetes and hypercholesterolaemia could play a role as biomarkers rather than to be a direct cause of arterial stiffening. Thus, the mechanisms through which the up‐regulated proteins shown here as associated with aortic stiffening operate on vascular disease are still incompletely worked out and deserve further investigation. Second, we still need to show that in vivo disruption of the AQP1/NFAT5 signalling, shown here as associated with aortic stiffening in diabetic mice, will mitigate in vivo vascular disease in response to high glucose. The absence of AQP1 and NFAT1 knockout mice having the comorbidities of diabetes and hypercholesterolaemia complicates the performance of this type of experiments. A possible solution might be the use of specific drugs for AQP1 and NFAT5, currently not available.

## 5 CONCLUSION

Diabetic and hypercholesterolaemic Ins^2+/Akita^: ApoE^−/−^ mice develop aortic stiffening, associated with up‐regulation of hypertonicity‐responsive gene such as AQ1 and NFAT5, along with genes implicated in early inflammation and atherosclerosis, such as VCAM‐1; cytoskeletal remodelling, such as F‐actin and ASMA; endothelial dysfunction, such as iNOS and eNOS; and ROS generation such as NADPH oxidase. These changes in gene expression may be important mechanisms leading to the development of arterial stiffening in diabetes and hypercholesterolaemia.

## CONFLICT OF INTEREST

PF is the founder and CEO of Pharmahungary Group, a group of R&D companies.

## AUTHOR CONTRIBUTIONS

Rosalinda Madonna designed and performed the experiments, analysed the data and wrote the manuscript. Vanessa Doria performed in vitro experiments and analysed the data. Nino Cocco analysed the data, provided advice. Anikó Görbe, Péter Ferdinandy, Yong‐Jian Geng and Sante Donato Pierdomenico analysed the data, provided advice support, corrected the final manuscript. Raffaele De Caterina analysed the data, provided advice and financial support, and reviewed and corrected the final manuscript.

## ETHICAL APPROVAL

All procedures were approved by the Institutional Ethics Committee for Animal Research (Protocol number 11/2012/CEISA/COM).

## Data Availability

Original data are available upon request from the journal.
